# Partially incorrect fossil data augment analyses of discrete trait evolution in living species

**DOI:** 10.1098/rsbl.2016.0392

**Published:** 2016-08

**Authors:** Mark N. Puttick

**Affiliations:** School of Earth Sciences, University of Bristol, Life Sciences Building, Tyndall Avenue, Bristol BS8 1TQ, UK

**Keywords:** ancestral states, fossils, discrete characters, phylogeny

## Abstract

Ancestral state reconstruction of discrete character traits is often vital when attempting to understand the origins and homology of traits in living species. The addition of fossils has been shown to alter our understanding of trait evolution in extant taxa, but researchers may avoid using fossils alongside extant species if only few are known, or if the designation of the trait of interest is uncertain. Here, I investigate the impacts of fossils and incorrectly coded fossils in the ancestral state reconstruction of discrete morphological characters under a likelihood model. Under simulated phylogenies and data, likelihood-based models are generally accurate when estimating ancestral node values. Analyses with combined fossil and extant data always outperform analyses with extant species alone, even when around one quarter of the fossil information is incorrect. These results are especially pronounced when model assumptions are violated, such as when there is a trend away from the root value. Fossil data are of particular importance when attempting to estimate the root node character state. Attempts should be made to include fossils in analysis of discrete traits under likelihood, even if there is uncertainty in the fossil trait data.

## 1. Introduction

Ancestral states reconstructions provide an important framework for understanding the origin, homology and timing of character state evolution [1]. Discrete character traits are of particular importance as they can be used to understand the acquisition of key traits during adaptive radiations, and are also used to model aspects of species' ecology and behaviour. In living species, ancestral state estimation can be used as an end in itself [2], but can also be important in downstream analyses such as understanding the relationship between traits [3]. In the past, ancestral state reconstruction has been used mostly for extant taxa [1], but fossil data can strengthen or even change our interpretations when used alongside neontological data [2,4–5].

Models of discrete character evolution assess the fit of models to the known data (tip values) when estimating ancestral states in a likelihood framework [1,6–8]. At a greater distance from the tips there is greater uncertainty as the observed data (tip values) is increasingly remote; this problem is acute in studies with only extant taxa. One solution to this is to include fossil information to reduce the distance between the tips and deeper nodes in the phylogeny. Recently, methodological advances have made it easier to include fossils alongside living species in dated phylogenies [9].

If possible, it is beneficial to always include fossils in ancestral state reconstructions [2,5]. Yet, fossils are often greatly outnumbered by extant species and there is likely to be a greater uncertainty in coding fossil trait data, especially for those traits not directly related to anatomy [10]. For example, a fossil species may easily be assigned an incorrect habitat, such as terrestrial rather than freshwater.

**Figure 1.**
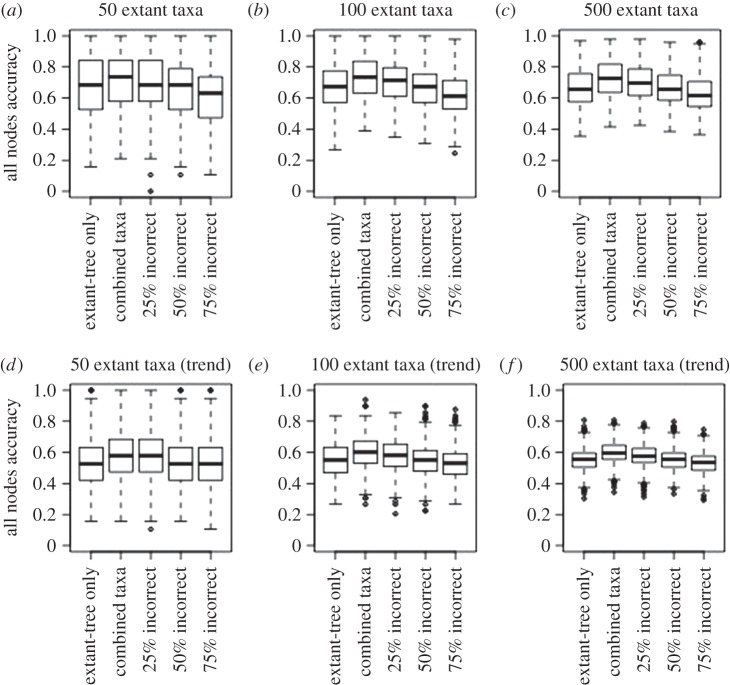
Distribution of the proportion of accurately reconstructed nodes throughout the phylogeny in datasets with four character states simulated with unequal rates (*a–c*) and a trend (*d–f*). Full results for all datasets are shown in the electronic supplementary material.

Here, I analyse the impact of including fossils and incorrectly coded fossils (figure 1) on the accuracy of ancestral state reconstructions under a likelihood model. In this study, incorrectly coded fossils represent tip states that were changed from the true state to a random incorrect state to mimic a scenario in which fossil data are available, but they provide inaccurate information. As expected, analyses with correctly coded fossils always improve the accuracy of reconstructions; this effect is more pronounced when simulations violate model assumptions. Surprisingly, even a proportion of incorrectly coded fossils can enhance the accuracy of ancestral estimation.

## 2. Material and methods

### (a) Phylogenies

I simulated phylogenies with a fixed number of extant species and a variable number of crown- and stem-group fossils. I simulated phylogenies in the R package TreeSim [11] with the following parameters: speciation rate (*λ*) = 1, extinction rate (*μ*) = 0.2 and fraction of extant sampled species = 1. These values were selected to simulate a scenario with a fixed number of extant taxa and a variable, but smaller number, of extinct taxa (electronic supplementary material, table S1) as may be typical of total-evidence phylogenies for clades such as certain vertebrates that have undergone a relatively recent radiation. To analyse the effects of sample size, I simulated phylogenies with 50, 100 and 500 extant tip species and a variable number of fossil species. I simulated 1000 individual trees with 50, 100 and 500 extant taxa, respectively.

### (b) Data simulation

I simulated discrete character data in R using custom written code [12] in a process similar to the Mk model of evolution with *k* states. For each dataset, individual characters were assumed to evolve independently of other characters. I simulated the evolution of characters with 2, 3, 4 and 5 states on each phylogeny. In each process of evolution, character rates were drawn from a uniform distribution between 0.5 and 2 (i.e. rates two times higher and lower than the base rate) to capture a wide range of different possible evolutionary scenarios for trait evolution.

I allowed character states to change in two distinct ways: in the equal rates model, I allowed character states to change to all other states with equal probability and selected a random character state at the root. In a second set of simulations, I ordered character states so that there was an overall trend to move away from root state to higher character state values. In each simulation, I set the root state to 0, and the matrix was coded so that a move to the adjacent higher state was favoured, and a move down character state values was less favoured. Thus, conditions were such as to simulate a trend model through time in which higher state values were more likely to be sampled at tips closer to the present.

I simulated discrete traits on a phylogeny and compared analyses with extant-only data and combined fossil and extant data. I randomly replaced some of the correct states at the fossil tips with other randomly selected states (that were always incorrect) in the simulation to produce incorrectly coded fossils and did this for 25%, 50% and 75% of the fossil tip states, respectively.

### (c) Ancestral state reconstruction

With the simulated data and phylogenies, I reconstructed marginal ancestral likelihoods in the R package APE [13]. I set the model of evolution for reconstructions as unordered (i.e. there is equal transition probability between states) which may be the most common scenario [14].

I judged a reconstruction to be accurate if the known trait value had the largest marginal likelihood under the reconstruction. As combined phylogenies of extant and extinct taxa have more nodes than the extant-only datasets, I used only data from the comparable nodes between the extant and combined phylogenies from each simulation for further analyses. Using the distributions of the proportion of accurately reconstructed nodes, I tested whether the distribution of values from the combined phylogenies were significantly greater than distributions from the extant phylogeny (Kolmogorov–Smirnov test).

## 3. Results

As the number of character states increases, there is a general trend for lower levels of accuracy in ancestral character state estimation in each of the simulations (electronic supplementary material, tables S2–S4).

In all simulations, the phylogeny with fossils significantly outperformed the extant-only simulations in accuracy of the node reconstructions (table 1; figure 1, electronic supplementary material tables S2–S5 and figures S1–S3). With 25% of fossils incorrectly coded, datasets were significantly more accurate than the extant-only data in the vast majority of cases. In most cases, the median accuracy is higher in the 25% incorrectly coded datasets (80%). Datasets with larger phylogenies and more character states are also significantly more accurate than the extant-only analysis when 50% of fossils are incorrectly coded.

**Table 1.** Median value and 95% quantile range of the accuracy (proportion of correctly reconstructed states) for all nodes throughout the phylogeny in the dataset with four character states and with 25%, 50% and 75% of fossil taxa coded incorrectly (for full results for all character states, see the electronic supplementary material, table S2).

		extant-only taxa	combined extant and extinct taxa	25% incorrect extinct taxa	50% incorrect extinct taxa	75% incorrect extinct taxa
four states	50 extant taxa	0.76 (0.59–0.92)	0.79 (0.65–0.92)	0.78 (0.61–0.92)	0.76 (0.59–0.88)	0.71 (0.53–0.86)
	100 extant taxa	0.78 (0.65–0.89)	0.81 (0.69–0.89)	0.78 (0.66–0.88)	0.75 (0.63–0.86)	0.71 (0.60–0.83)
	500 extant taxa	0.79 (0.73–0.83)	0.81 (0.76–0.85)	0.78 (0.73–0.83)	0.75 (0.69–0.80)	0.71 (0.65–0.76)
	50 extant taxa (trend)	0.65 (0.50–0.82)	0.71 (0.55–0.86)	0.63 (0.51–0.84)	0.65 (0.49–0.80)	0.61 (0.47–0.80)
	100 extant taxa (trend)	0.65 (0.54–0.78)	0.72 (0.59–0.82)	0.68 (0.57–0.79)	0.65 (0.54–0.77)	0.62 (0.49–0.74)
	500 extant taxa (trend)	0.66 (0.60–0.71)	0.71 (0.65–0.76)	0.68 (0.63–0.73)	0.65 (0.59–0.71)	0.62 (0.56–0.67)

**Figure 2.**
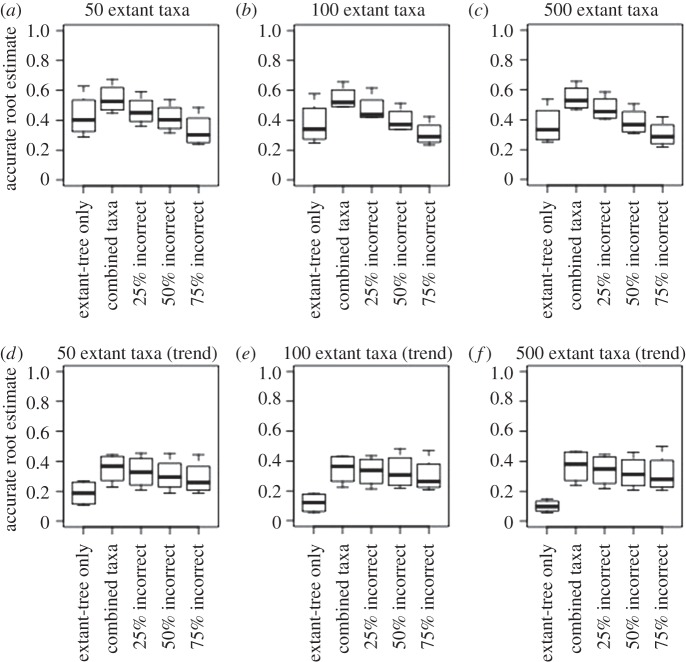
The distribution of the proportion of accurately reconstructed root nodes summarizing results from analyses with all character states simulated with unequal rates (*a–c*) and a trend (*d–f*).

For root node reconstruction, phylogenies with fossils outperform extant-only data in every dataset (table 2 and figure 2). The number of correct root node character state estimates is higher in all datasets with 25% incorrectly coded fossils (electronic supplementary material, table S4 and figures S4–S7), in datasets with 50% incorrectly coded fossil states (88% datasets) and 75% incorrectly coded fossil states (54% datasets). Median estimates of the correct marginal likelihood are higher for most of the 25% incorrectly coded fossil dataset compared with the extant-only dataset (54%). Phylogenies with stem-group fossils (i.e. outgroups to living taxa) have a slightly better chance of accurately reconstructing root node character states (approx. 5% improved across all datasets). In datasets with incorrect data and an inaccurate root node estimate, the oldest incorrect fossils are more likely to be closer to the root (approx. 2% tree length) compared with when the root node reconstruction is accurate (see the electronic supplementary material, table S5).

**Table 2.** Median value and 95% quantile range of the accuracy (proportion of correctly reconstructed states) of the root node in all datasets and with 25%, 50% and 75% of fossil taxa coded incorrectly (for full results for all character states, see the electronic supplementary material, table S3).

		extant-only taxa	combined extant and extinct taxa	25% incorrect extinct taxa	50% incorrect extinct taxa	75% incorrect extinct taxa
four states	50 extant taxa	0.48 (0.09–0.61)	0.49 (0.071–0.89)	0.49 (0.14–0.85)	0.49 (0.17–0.77)	0.50 (0.21–0.76)
	100 extant taxa	0.33 (0.27–0.35)	0.34 (0.16–0.79)	0.33 (0.16–0.73)	0.33 (0.15–0.64)	0.32 (0.15–0.55)
	500 extant taxa	0.25 (0.23–0.25)	0.24 (0.13–0.68)	0.24 (0.13–0.59)	0.25 (0.13–0.50)	0.25 (0.13–0.42)
	50 extant taxa (trend)	0.20 (0.19–0.20)	0.19 (0.12–0.54)	0.19 (0.11–0.46)	0.19 (0.11–0.39)	0.19 (0.12–0.34)
	100 extant taxa (trend)	0.50 (0.47–0.56)	0.56 (0.260–0.95)	0.52 (0.25–0.88)	0.49 (0.22–0.77)	0.49 (0.22–0.72)
	500 extant taxa (trend)	0.33 (0.29–0.41)	0.39 (0.16–0.94)	0.35 (0.15–0.85)	0.33 (0.13–0.71)	0.32 (0.13–0.89)

## 4. Discussion

Fossils can increase our understanding of past change, and surprisingly, they still have positive effects even when some of the information they provide is incorrect. Therefore, it seems prudent to include fossils whenever possible when reconstructing ancestral states with extant species [2].

As expected, trees with all simulated fossils correctly coded always outperform extant-only datasets. For simulations with no trend, both extant-only and phylogenies with fossils indicate a high accuracy of ancestral state estimation for all nodes (table 1 and figure 1). However, fossils still outperform extant-only data when some are incorrectly coded. Generally, only when 75% of fossils are incorrect is there a significant difference with fossils performing worse than extant-only data (figure 1). If model assumptions are particularly violated, as seen in the simulation with a trend, fossil information becomes increasingly important, especially for the root node (table 2 and figure 2).

In this study, we have only considered ancestral state reconstructions of discrete traits in a likelihood framework [1,6]. A range of alternative methods are available to elucidate past character evolution, stochastic character mapping [15] and threshold models [16,17]. Here, I concentrate upon the impact of fossils on the widely used likelihood model of discrete character evolution [13] and not on a comparison of the effectiveness of alternative methods. Additionally, the main focus here is on the accuracy of methods, but there is also evidence fossils increase the precision of marginal likelihood estimates of ancestral nodes (electronic supplementary material, table S4). Another caveat in this study is that the tree topology and divergence times are assumed to be known without error. These potential errors can add complexities to ancestral state reconstruction as fossil data can impact both of these. However, it is generally recommended that fossils are included in analyses whenever possible [2,5]. This could be in the form of including fossil data in morphological matrices for the analysis of topology and divergence time estimation (e.g. [9]), or by fossil data acting as priors on internal nodes for studies of trait evolution [18].

## 5. Conclusion

Analysis of discrete trait evolution is key to understanding patterns of character change and diversity of extant taxa. However, living species only represent a small fraction of diversity, and so it is important to include some fossil information to better understand past change, even if some of this information may be incorrect. It is shown here that even uncertain fossil information is not detrimental to ancestral state reconstructions, and the simulations here demonstrate that even incorrect fossils are better than no fossils at all.

## Supplementary Material

Supplementary Materials

## Data accessibility

The R scripts for generating the phylogenies and data are available on Dryad (http://dx.doi.org/10.5061/dryad.v66b2).
