# Supplementary material for: Partially incorrect fossil data augment analyses of discrete trait evolution in living species
Source: Biol Lett. 2016 Aug;12(8):20160392. doi: 10.1098/rsbl.2016.0392 (PMC5014033; doi:10.1098/rsbl.2016.0392)
Supplement: Supplementary Materials [file rsbl20160392supp1.docx]

**Supplementary Materials**

**Supplementary Table S1.** The quantiles for the number of fossil taxa in each of the 1000 distribution of phylogenies with 50, 100, and 500 taxa respectively.

|  | **0** | **0.25** | **0.5** | **0.75** | **1** |
| --- | --- | --- | --- | --- | --- |
| **fifty extant taxa** | 2 | 9 | 12 | 16 | 32 |
| **one hundred extant taxa** | 5 | 20 | 24 | 29 | 50 |
| **five hundred extant taxa** | 86 | 114 | 125 | 136 | 177 |

|  |  | **extant-only taxa** | **combined extant**  **and extinct taxa** | **25% incorrect**  **extinct taxa** | **50% incorrect**  **extinct taxa** | **75% incorrect**  **extinct taxa** |
| --- | --- | --- | --- | --- | --- | --- |
| **two states** | **50 extant taxa** | 0.796 (0.592-0.959) | 0.816 (0.633-0.98) | 0.796 (0.592-0.959) | 0.755 (0.571-0.918) | 0.714 (0.55-0.898) |
|  | **100 extant taxa** | 0.778 (0.636-0.929) | 0.818 (0.677-0.949) | 0.788 (0.636-0.929) | 0.747 (0.616-0.889) | 0.707 (0.586-0.848) |
|  | **500 extant taxa** | 0.774 (0.683-0.902) | 0.814 (0.721-0.93) | 0.782 (0.695-0.9) | 0.745 (0.667-0.846) | 0.707 (0.633-0.796) |
|  | **50 extant taxa (trend)** | 0.714 (0.509-0.939) | 0.755 (0.551-0.959) | 0.735 (0.531-0.939) | 0.694 (0.509-0.898) | 0.653 (0.469-0.857) |
|  | **100 extant taxa (trend)** | 0.707 (0.535-0.909) | 0.768 (0.596-0.929) | 0.727 (0.565-0.909) | 0.687 (0.525-0.879) | 0.646 (0.505-0.838) |
|  | **500 extant taxa (trend)** | 0.694 (0.585-0.868) | 0.753 (0.627-0.908) | 0.719 (0.605-0.878) | 0.683 (0.579-0.838) | 0.647 (0.549-0.78) |
| **three states** | **50 extant taxa** | 0.673 (0.449-0.898) | 0.735 (0.51-0.939) | 0.714 (0.49-0.918) | 0.673 (0.449-0.898) | 0.612 (0.429-0.857) |
|  | **100 extant taxa** | 0.657 (0.485-0.879) | 0.727 (0.535-0.919) | 0.697 (0.515-0.899) | 0.657 (0.485-0.849) | 0.616 (0.465-0.808) |
|  | **500 extant taxa** | 0.656 (0.531-0.854) | 0.723 (0.593-0.904) | 0.69 (0.565-0.874) | 0.657 (0.541-0.83) | 0.616 (0.509-0.788) |
|  | **50 extant taxa (trend)** | 0.673 (0.429-0.918) | 0.735 (0.51-0.939) | 0.694 (0.469-0.918) | 0.653 (0.449-0.898) | 0.612 (0.388-0.857) |
|  | **100 extant taxa (trend)** | 0.641 (0.455-0.869) | 0.707 (0.515-0.919) | 0.677 (0.494-0.889) | 0.646 (0.465-0.859) | 0.606 (0.434-0.818) |
|  | **500 extant taxa (trend)** | 0.633 (0.505-0.846) | 0.697 (0.559-0.894) | 0.667 (0.533-0.868) | 0.635 (0.513-0.834) | 0.597 (0.483-0.78) |
| **four states** | **50 extant taxa** | 0.755 (0.592-0.918) | 0.796 (0.653-0.918) | 0.776 (0.612-0.918) | 0.755 (0.592-0.878) | 0.714 (0.531-0.857) |
|  | **100 extant taxa** | 0.778 (0.646-0.889) | 0.808 (0.687-0.899) | 0.778 (0.657-0.879) | 0.747 (0.626-0.859) | 0.707 (0.596-0.828) |
|  | **500 extant taxa** | 0.786 (0.731-0.834) | 0.81 (0.762-0.852) | 0.784 (0.733-0.832) | 0.747 (0.693-0.8) | 0.707 (0.647-0.762) |
|  | **50 extant taxa (trend)** | 0.653 (0.49-0.816) | 0.714 (0.551-0.857) | 0.673 (0.51-0.837) | 0.653 (0.49-0.796) | 0.612 (0.469-0.796) |
|  | **100 extant taxa (trend)** | 0.652 (0.535-0.778) | 0.717 (0.586-0.818) | 0.677 (0.565-0.798) | 0.646 (0.535-0.768) | 0.616 (0.495-0.737) |
|  | **500 extant taxa (trend)** | 0.656 (0.603-0.713) | 0.707 (0.653-0.758) | 0.679 (0.625-0.731) | 0.649 (0.587-0.705) | 0.615 (0.563-0.667) |
| **five states** | **50 extant taxa** | 0.551 (0.388-0.714) | 0.602 (0.429-0.755) | 0.582 (0.408-0.735) | 0.551 (0.388-0.714) | 0.531 (0.367-0.694) |
|  | **100 extant taxa** | 0.556 (0.434-0.677) | 0.596 (0.485-0.707) | 0.576 (0.465-0.687) | 0.556 (0.434-0.667) | 0.535 (0.414-0.646) |
|  | **500 extant taxa** | 0.549 (0.499-0.605) | 0.597 (0.547-0.645) | 0.575 (0.525-0.625) | 0.551 (0.497-0.599) | 0.527 (0.473-0.579) |
|  | **50 extant taxa (trend)** | 0.469 (0.327-0.633) | 0.51 (0.347-0.673) | 0.49 (0.346-0.653) | 0.469 (0.327-0.633) | 0.449 (0.306-0.612) |
|  | **100 extant taxa (trend)** | 0.475 (0.364-0.586) | 0.515 (0.404-0.626) | 0.495 (0.384-0.606) | 0.475 (0.364-0.586) | 0.465 (0.343-0.576) |
|  | **500 extant taxa (trend)** | 0.475 (0.427-0.521) | 0.515 (0.467-0.565) | 0.497 (0.449-0.547) | 0.477 (0.429-0.527) | 0.455 (0.407-0.505) |

**Supplementary Table S2.** Overall fraction of accurately reconstructed nodes for 1000 phylogenies for the simulated data and simulated data with a trend. The values represent the median and 95% quantile range for each set of 1000 phylogenies from the simulated data (with and without a trend).

|  |  | **extant-only taxa** | **combined extant**  **and extinct taxa** | **25% incorrect**  **extinct taxa** | **50% incorrect**  **extinct taxa** | **75% incorrect**  **extinct taxa** |
| --- | --- | --- | --- | --- | --- | --- |
| **two states** | **50 extant taxa** | 0.5 (0.377-0.738) | 0.557 (0.264-0.954) | 0.515 (0.225-0.905) | 0.5 (0.204-0.799) | 0.498 (0.153-0.734) |
|  | **100 extant taxa** | 0.333 (0.234-0.585) | 0.384 (0.149-0.938) | 0.338 (0.133-0.869) | 0.333 (0.124-0.769) | 0.325 (0.11-0.586) |
|  | **500 extant taxa** | 0.25 (0.162-0.509) | 0.28 (0.106-0.92) | 0.252 (0.103-0.857) | 0.249 (0.098-0.752) | 0.241 (0.087-0.517) |
|  | **50 extant taxa (trend)** | 0.2 (0.131-0.45) | 0.247 (0.078-0.927) | 0.209 (0.077-0.846) | 0.198 (0.076-0.717) | 0.192 (0.062-0.517) |
|  | **100 extant taxa (trend)** | 0.48 (0.064-0.605) | 0.484 (0.08-0.918) | 0.493 (0.118-0.862) | 0.5 (0.126-0.782) | 0.5 (0.155-0.735) |
|  | **500 extant taxa (trend)** | 0.332 (0.205-0.354) | 0.332 (0.123-0.759) | 0.331 (0.137-0.685) | 0.33 (0.138-0.629) | 0.325 (0.143-0.534) |
| **three states** | **50 extant taxa** | 0.25 (0.177-0.255) | 0.245 (0.122-0.681) | 0.246 (0.118-0.636) | 0.244 (0.114-0.509) | 0.245 (0.117-0.404) |
|  | **100 extant taxa** | 0.2 (0.167-0.2) | 0.197 (0.108-0.529) | 0.197 (0.108-0.437) | 0.198 (0.112-0.393) | 0.199 (0.102-0.333) |
|  | **500 extant taxa** | 0.5 (0.417-0.675) | 0.548 (0.272-0.945) | 0.513 (0.247-0.872) | 0.5 (0.196-0.773) | 0.496 (0.188-0.732) |
|  | **50 extant taxa (trend)** | 0.333 (0.239-0.555) | 0.393 (0.153-0.935) | 0.349 (0.151-0.883) | 0.332 (0.126-0.747) | 0.323 (0.105-0.618) |
|  | **100 extant taxa (trend)** | 0.25 (0.186-0.419) | 0.28 (0.101-0.934) | 0.25 (0.095-0.833) | 0.247 (0.096-0.715) | 0.235 (0.079-0.548) |
|  | **500 extant taxa (trend)** | 0.2 (0.143-0.381) | 0.231 (0.096-0.927) | 0.204 (0.088-0.876) | 0.195 (0.079-0.744) | 0.19 (0.063-0.518) |
| **four states** | **50 extant taxa** | 0.479 (0.095-0.607) | 0.489 (0.071-0.889) | 0.492 (0.139-0.846) | 0.498 (0.171-0.768) | 0.5 (0.205-0.761) |
|  | **100 extant taxa** | 0.332 (0.272-0.348) | 0.338 (0.161-0.796) | 0.332 (0.155-0.73) | 0.327 (0.148-0.637) | 0.324 (0.145-0.545) |
|  | **500 extant taxa** | 0.25 (0.225-0.251) | 0.242 (0.13-0.68) | 0.243 (0.13-0.588) | 0.245 (0.131-0.503) | 0.245 (0.125-0.412) |
|  | **50 extant taxa (trend)** | 0.2 (0.193-0.2) | 0.196 (0.115-0.542) | 0.196 (0.112-0.455) | 0.197 (0.109-0.393) | 0.198 (0.118-0.343) |
|  | **100 extant taxa (trend)** | 0.5 (0.466-0.559) | 0.564 (0.259-0.947) | 0.517 (0.249-0.876) | 0.499 (0.219-0.768) | 0.494 (0.217-0.712) |
|  | **500 extant taxa (trend)** | 0.333 (0.298-0.409) | 0.398 (0.164-0.936) | 0.35 (0.146-0.85) | 0.33 (0.133-0.713) | 0.324 (0.132-0.589) |
| **five states** | **50 extant taxa** | 0.25 (0.218-0.308) | 0.301 (0.118-0.92) | 0.259 (0.097-0.821) | 0.245 (0.088-0.679) | 0.234 (0.083-0.537) |
|  | **100 extant taxa** | 0.2 (0.167-0.277) | 0.249 (0.091-0.907) | 0.215 (0.089-0.815) | 0.196 (0.083-0.681) | 0.184 (0.068-0.447) |
|  | **500 extant taxa** | 0.475 (0.202-0.592) | 0.464 (0.118-0.89) | 0.494 (0.192-0.844) | 0.5 (0.239-0.766) | 0.5 (0.221-0.698) |
|  | **50 extant taxa (trend)** | 0.333 (0.322-0.336) | 0.341 (0.175-0.8) | 0.331 (0.172-0.716) | 0.329 (0.168-0.654) | 0.323 (0.145-0.521) |
|  | **100 extant taxa (trend)** | 0.25 (0.248-0.25) | 0.245 (0.135-0.651) | 0.244 (0.142-0.573) | 0.244 (0.147-0.497) | 0.245 (0.147-0.416) |
|  | **500 extant taxa (trend)** | 0.2 (0.2-0.2) | 0.195 (0.127-0.552) | 0.195 (0.127-0.477) | 0.197 (0.121-0.399) | 0.196 (0.119-0.313) |

**Supplementary Table S3.** Median value and 95% quantile range of the estimated marginal likelihood of the correct character state at the root node for all datasets.

|  | **Accurate nodes** | | | | | **Marginal likelihood** | | | | |
| --- | --- | --- | --- | --- | --- | --- | --- | --- | --- | --- |
|  | **Full Tree** | **Extant Only** | **25% Incorrect** | **50% Incorrect** | **75% Incorrect** | **Full Tree** | **Extant Only** | **25% Incorrect** | **50% Incorrect** | **75% Incorrect** |
| **Two Characters 50 Taxa** | 0.819 | 0.784 (0.334) | 0.79 (0.281) | 0.752 (0.648) | 0.714 (1.019) | 0.809 | 0.753 (0.217) | 0.763 (0.178) | 0.719 (0.351) | 0.681 (0.503) |
| **Two Characters 100 Taxa** | 0.818 | 0.78 (0.451) | 0.786 (0.379) | 0.749 (0.829) | 0.711 (1.282) | 0.816 | 0.751 (0.259) | 0.766 (0.199) | 0.72 (0.383) | 0.678 (0.547) |
| **Two Characters 500 Taxa** | 0.819 | 0.781 (0.583) | 0.788 (0.469) | 0.75 (1.046) | 0.709 (1.664) | 0.825 | 0.754 (0.286) | 0.772 (0.212) | 0.719 (0.426) | 0.671 (0.618) |
| **Two Characters 50 Taxa Trend** | 0.758 | 0.715 (0.344) | 0.727 (0.247) | 0.696 (0.498) | 0.657 (0.81) | 0.731 | 0.656 (0.243) | 0.682 (0.158) | 0.635 (0.31) | 0.586 (0.47) |
| **Two Characters 100 Taxa Trend** | 0.765 | 0.713 (0.491) | 0.735 (0.281) | 0.696 (0.651) | 0.659 (1.002) | 0.739 | 0.654 (0.278) | 0.691 (0.159) | 0.635 (0.342) | 0.586 (0.502) |
| **Two Characters 500 Taxa Trend** | 0.761 | 0.709 (0.588) | 0.73 (0.347) | 0.695 (0.755) | 0.655 (1.203) | 0.741 | 0.643 (0.323) | 0.686 (0.182) | 0.627 (0.376) | 0.57 (0.564) |
| **Three Characters 50 Taxa** | 0.731 | 0.672 (0.447) | 0.703 (0.217) | 0.669 (0.475) | 0.63 (0.769) | 0.678 | 0.589 (0.268) | 0.631 (0.14) | 0.579 (0.297) | 0.53 (0.444) |
| **Three Characters 100 Taxa** | 0.728 | 0.67 (0.499) | 0.699 (0.253) | 0.666 (0.536) | 0.628 (0.859) | 0.683 | 0.592 (0.273) | 0.638 (0.135) | 0.583 (0.298) | 0.53 (0.457) |
| **Three Characters 500 Taxa** | 0.732 | 0.674 (0.602) | 0.702 (0.306) | 0.668 (0.662) | 0.629 (1.058) | 0.702 | 0.594 (0.327) | 0.647 (0.166) | 0.588 (0.346) | 0.527 (0.529) |
| **Three Characters 50 Taxa Trend** | 0.726 | 0.668 (0.431) | 0.696 (0.226) | 0.663 (0.47) | 0.621 (0.789) | 0.684 | 0.583 (0.289) | 0.635 (0.141) | 0.584 (0.287) | 0.525 (0.457) |
| **Three Characters 100 Taxa Trend** | 0.713 | 0.651 (0.491) | 0.684 (0.228) | 0.652 (0.489) | 0.612 (0.804) | 0.664 | 0.564 (0.284) | 0.615 (0.139) | 0.563 (0.287) | 0.505 (0.453) |
| **Three Characters 500 Taxa Trend** | 0.713 | 0.651 (0.585) | 0.683 (0.281) | 0.651 (0.583) | 0.612 (0.946) | 0.669 | 0.561 (0.311) | 0.616 (0.151) | 0.561 (0.311) | 0.502 (0.48) |
| **Four Characters 50 Taxa** | 0.794 | 0.759 (0.399) | 0.773 (0.234) | 0.742 (0.592) | 0.706 (1.008) | 0.823 | 0.777 (0.165) | 0.77 (0.188) | 0.716 (0.382) | 0.68 (0.509) |
| **Four Characters 100 Taxa** | 0.801 | 0.772 (0.456) | 0.776 (0.399) | 0.745 (0.89) | 0.711 (1.423) | 0.855 | 0.799 (0.198) | 0.79 (0.23) | 0.723 (0.468) | 0.673 (0.644) |
| **Four Characters 500 Taxa** | 0.808 | 0.784 (0.839) | 0.782 (0.918) | 0.747 (2.146) | 0.706 (3.587) | 0.891 | 0.825 (0.233) | 0.807 (0.297) | 0.723 (0.598) | 0.66 (0.822) |
| **Four Characters 50 Taxa Trend** | 0.708 | 0.655 (0.572) | 0.683 (0.276) | 0.65 (0.623) | 0.616 (0.984) | 0.641 | 0.557 (0.271) | 0.594 (0.15) | 0.551 (0.29) | 0.504 (0.443) |
| **Four Characters 100 Taxa Trend** | 0.709 | 0.656 (0.766) | 0.679 (0.436) | 0.652 (0.827) | 0.618 (1.317) | 0.648 | 0.545 (0.335) | 0.587 (0.199) | 0.54 (0.353) | 0.495 (0.5) |
| **Four Characters 500 Taxa Trend** | 0.707 | 0.657 (1.595) | 0.679 (0.877) | 0.649 (1.834) | 0.615 (2.883) | 0.641 | 0.515 (0.416) | 0.575 (0.218) | 0.519 (0.403) | 0.471 (0.561) |
| **Five Characters 50 Taxa** | 0.597 | 0.551 (0.457) | 0.576 (0.21) | 0.551 (0.458) | 0.528 (0.681) | 0.456 | 0.382 (0.227) | 0.424 (0.098) | 0.39 (0.202) | 0.364 (0.283) |
| **Five Characters 100 Taxa** | 0.599 | 0.552 (0.678) | 0.578 (0.308) | 0.553 (0.665) | 0.531 (0.978) | 0.441 | 0.363 (0.243) | 0.407 (0.105) | 0.371 (0.216) | 0.348 (0.289) |
| **Five Characters 500 Taxa** | 0.597 | 0.551 (1.498) | 0.574 (0.729) | 0.55 (1.515) | 0.526 (2.29) | 0.425 | 0.342 (0.261) | 0.389 (0.113) | 0.358 (0.209) | 0.331 (0.294) |
| **Five Characters 50 Taxa Trend** | 0.511 | 0.464 (0.49) | 0.494 (0.182) | 0.474 (0.385) | 0.454 (0.598) | 0.315 | 0.264 (0.158) | 0.295 (0.063) | 0.276 (0.121) | 0.261 (0.168) |
| **Five Characters 100 Taxa Trend** | 0.517 | 0.473 (0.63) | 0.498 (0.267) | 0.479 (0.547) | 0.461 (0.807) | 0.308 | 0.253 (0.17) | 0.286 (0.066) | 0.267 (0.127) | 0.251 (0.176) |
| **Five Characters 500 Taxa Trend** | 0.515 | 0.474 (1.367) | 0.497 (0.616) | 0.477 (1.277) | 0.456 (1.993) | 0.3 | 0.241 (0.187) | 0.277 (0.073) | 0.257 (0.137) | 0.24 (0.187) |

**Supplementary Table S4.** The accuracy of node reconstructions for all trees and datasets. For “accurate nodes” the values represent the mean fraction of nodes reconstructed accurately. For the “Marginal likelihood” , the mean marginal likelihood is reported for every reconstructed node throughout the tree. For both sets of data, the values in brackets represent the number of standard deviations a value is from the estimates from the full tree of extant and fossil data).

|  |  | **p values** | | | |
| --- | --- | --- | --- | --- | --- |
|  |  | **Full tree** | **25% wrong** | **50% wrong** | **75% wrong** |
| **two states** | **50 extant taxa** | <0.001 | 0.236 | 1.000 | 1.000 |
|  | **100 extant taxa** | <0.001 | 0.027 | 0.996 | 1.000 |
|  | **500 extant taxa** | <0.001 | 0.015 | 0.991 | 1.000 |
|  | **50 extant taxa (trend)** | <0.001 | 0.024 | 0.952 | 1.000 |
|  | **100 extant taxa (trend)** | <0.001 | <0.001 | 0.996 | 1.000 |
|  | **500 extant taxa (trend)** | <0.001 | <0.001 | 1.000 | 1.000 |
| **three states** | **50 extant taxa** | <0.001 | <0.001 | 0.822 | 1.000 |
|  | **100 extant taxa** | <0.001 | <0.001 | 0.562 | 0.999 |
|  | **500 extant taxa** | <0.001 | <0.001 | 0.670 | 1.000 |
|  | **50 extant taxa (trend)** | <0.001 | 0.002 | 0.749 | 0.996 |
|  | **100 extant taxa (trend)** | <0.001 | <0.001 | 0.294 | 0.999 |
|  | **500 extant taxa (trend)** | <0.001 | <0.001 | 0.431 | 1.000 |
| **four states** | **50 extant taxa** | <0.001 | 0.007 | 0.991 | 1.000 |
|  | **100 extant taxa** | <0.001 | 0.110 | 1.000 | 1.000 |
|  | **500 extant taxa** | <0.001 | 0.905 | 1.000 | 1.000 |
|  | **50 extant taxa (trend)** | <0.001 | <0.001 | 0.616 | 1.000 |
|  | **100 extant taxa (trend)** | <0.001 | <0.001 | 0.991 | 1.000 |
|  | **500 extant taxa (trend)** | <0.001 | <0.001 | 0.984 | 1.000 |
| **five states** | **50 extant taxa** | <0.001 | <0.001 | 0.774 | 0.999 |
|  | **100 extant taxa** | <0.001 | <0.001 | 0.482 | 1.000 |
|  | **500 extant taxa** | <0.001 | <0.001 | 0.723 | 0.999 |
|  | **50 extant taxa (trend)** | <0.001 | <0.001 | 0.060 | 0.991 |
|  | **100 extant taxa (trend)** | <0.001 | <0.001 | 0.157 | 0.996 |
|  | **500 extant taxa (trend)** | <0.001 | <0.001 | 0.035 | 1.000 |

**Supplementary Table S5.** p Values from Kolmogorov-Smirnov tests showing the significance of tests between the node accuracy of the extant-only analyses and the combined extinct and extant data, and data with 25%, 50%, and 75% coded incorrectly respectively. The test were aimed at establishing whether nodes accuracy distributions from the combined fossil and extant datasets were greater than the extant-only data.

|  | | **25% fossils wrong** | | | **50% fossils wrong** | | **75% fossils wrong** | |
| --- | --- | --- | --- | --- | --- | --- | --- | --- |
|  |  | **Median distance from root of incorrect fossil** | | | | | | |
|  |  | Accurate root estimate | Inaccurate root estimate | Accurate root estimate | | Inaccurate root estimate | Accurate root estimate | Inaccurate root estimate |
| **two states** | **50 extant taxa** | 0.413  (0.083-0.889) | 0.451  (0.101-0.935) | 0.556 (0.205-0.927) | | 0.649 (0.211-0.96) | 0.635 (0.296-0.956) | 0.721 (0.307-0.974) |
|  | **100 extant taxa** | 0.518 (0.209-0.877) | 0.542 (0.189-0.942) | 0.641 (0.334-0.936) | | 0.726 (0.352-0.971) | 0.699 (0.422-0.968) | 0.764 (0.444-0.967) |
|  | **500 extant taxa** | 0.636 (0.381-0.912) | 0.694 (0.37-0.94) | 0.728 (0.491-0.957) | | 0.794 (0.515-0.974) | 0.781 (0.581-0.966) | 0.846 (0.601-0.979) |
|  | **50 extant taxa (trend)** | 0.412 (0.081-0.865) | 0.468 (0.117-0.945) | 0.559 (0.194-0.9) | | 0.628 (0.244-0.963) | 0.635 (0.28-0.953) | 0.707 (0.308-0.979) |
|  | **100 extant taxa (trend)** | 0.505 (0.181-0.876) | 0.542 (0.195-0.943) | 0.633 (0.297-0.938) | | 0.702 (0.354-0.966) | 0.715 (0.45-0.96) | 0.76 (0.445-0.973) |
|  | **500 extant taxa (trend)** | 0.651 (0.386-0.934) | 0.689 (0.391-0.937) | 0.729 (0.505-0.943) | | 0.788 (0.519-0.971) | 0.786 (0.583-0.966) | 0.822 (0.582-0.978) |
| **three states** | **50 extant taxa** | 0.382 (0.075-0.882) | 0.449 (0.103-0.908) | 0.56 (0.183-0.924) | | 0.628 (0.229-0.956) | 0.6 (0.26-0.929) | 0.716 (0.323-0.975) |
|  | **100 extant taxa** | 0.518 (0.196-0.853) | 0.544 (0.205-0.929) | 0.624 (0.342-0.927) | | 0.693 (0.333-0.967) | 0.717 (0.418-0.95) | 0.755 (0.435-0.973) |
|  | **500 extant taxa** | 0.624 (0.383-0.911) | 0.662 (0.397-0.937) | 0.735 (0.495-0.938) | | 0.783 (0.519-0.973) | 0.782 (0.529-0.97) | 0.829 (0.605-0.978) |
|  | **50 extant taxa (trend)** | 0.42 (0.103-0.844) | 0.437 (0.084-0.887) | 0.556 (0.224-0.913) | | 0.62 (0.224-0.968) | 0.591 (0.269-0.943) | 0.705 (0.328-0.979) |
|  | **100 extant taxa (trend)** | 0.502 (0.209-0.863) | 0.538 (0.183-0.947) | 0.633 (0.34-0.924) | | 0.697 (0.341-0.963) | 0.709 (0.42-0.936) | 0.756 (0.443-0.972) |
|  | **500 extant taxa (trend)** | 0.624 (0.366-0.895) | 0.656 (0.366-0.948) | 0.723 (0.485-0.966) | | 0.78 (0.527-0.957) | 0.781 (0.567-0.96) | 0.829 (0.604-0.978) |
| **four states** | **50 extant taxa** | 0.463 (0.127-0.884) | 0.42 (0.079-0.899) | 0.604 (0.236-0.934) | | 0.579 (0.198-0.944) | 0.69 (0.362-0.948) | 0.702 (0.284-0.983) |
|  | **100 extant taxa** | 0.547 (0.202-0.899) | 0.522 (0.214-0.921) | 0.676 (0.362-0.938) | | 0.674 (0.324-0.966) | 0.729 (0.438-0.962) | 0.774 (0.429-0.972) |
|  | **500 extant taxa** | 0.666 (0.387-0.937) | 0.648 (0.398-0.95) | 0.754 (0.508-0.957) | | 0.758 (0.512-0.964) | 0.802 (0.563-0.97) | 0.846 (0.59-0.979) |
|  | **50 extant taxa (trend)** | 0.441 (0.099-0.879) | 0.429 (0.095-0.887) | 0.589 (0.234-0.958) | | 0.617 (0.181-0.951) | 0.674 (0.299-0.951) | 0.696 (0.317-0.969) |
|  | **100 extant taxa (trend)** | 0.509 (0.212-0.862) | 0.542 (0.186-0.932) | 0.643 (0.299-0.926) | | 0.686 (0.343-0.966) | 0.723 (0.436-0.956) | 0.757 (0.403-0.97) |
|  | **500 extant taxa (trend)** | 0.66 (0.385-0.935) | 0.674 (0.39-0.945) | 0.75 (0.513-0.954) | | 0.778 (0.512-0.97) | 0.798 (0.596-0.97) | 0.834 (0.584-0.979) |
| **five states** | **50 extant taxa** | 0.462 (0.095-0.894) | 0.415 (0.081-0.887) | 0.556 (0.228-0.915) | | 0.591 (0.192-0.951) | 0.696 (0.338-0.951) | 0.694 (0.295-0.979) |
|  | **100 extant taxa** | 0.56 (0.252-0.91) | 0.529 (0.182-0.934) | 0.686 (0.342-0.969) | | 0.663 (0.334-0.956) | 0.734 (0.475-0.974) | 0.751 (0.429-0.969) |
|  | **500 extant taxa** | 0.662 (0.381-0.922) | 0.668 (0.403-0.955) | 0.756 (0.496-0.969) | | 0.777 (0.52-0.971) | 0.806 (0.578-0.972) | 0.817 (0.584-0.977) |
|  | **50 extant taxa (trend)** | 0.501 (0.155-0.883) | 0.443 (0.084-0.911) | 0.602 (0.251-0.968) | | 0.58 (0.207-0.943) | 0.682 (0.365-0.963) | 0.689 (0.3-0.97) |
|  | **100 extant taxa (trend)** | 0.603 (0.164-0.876) | 0.517 (0.201-0.92) | 0.695 (0.355-0.944) | | 0.676 (0.318-0.963) | 0.764 (0.442-0.973) | 0.743 (0.432-0.97) |
|  | **500 extant taxa (trend)** | 0.671 (0.374-0.956) | 0.657 (0.397-0.933) | 0.744 (0.504-0.935) | | 0.762 (0.522-0.971) | 0.804 (0.603-0.978) | 0.824 (0.58-0.977) |

**Supplementary Table S6.** The median distance and 95% quantile range of the distance from the root of incorrectly-coded fossils in datasets that accurately and inaccurately reconstructed the root node. Here, accuracy is judge on the correct state having the highest marginal likelihood in reconstructions.


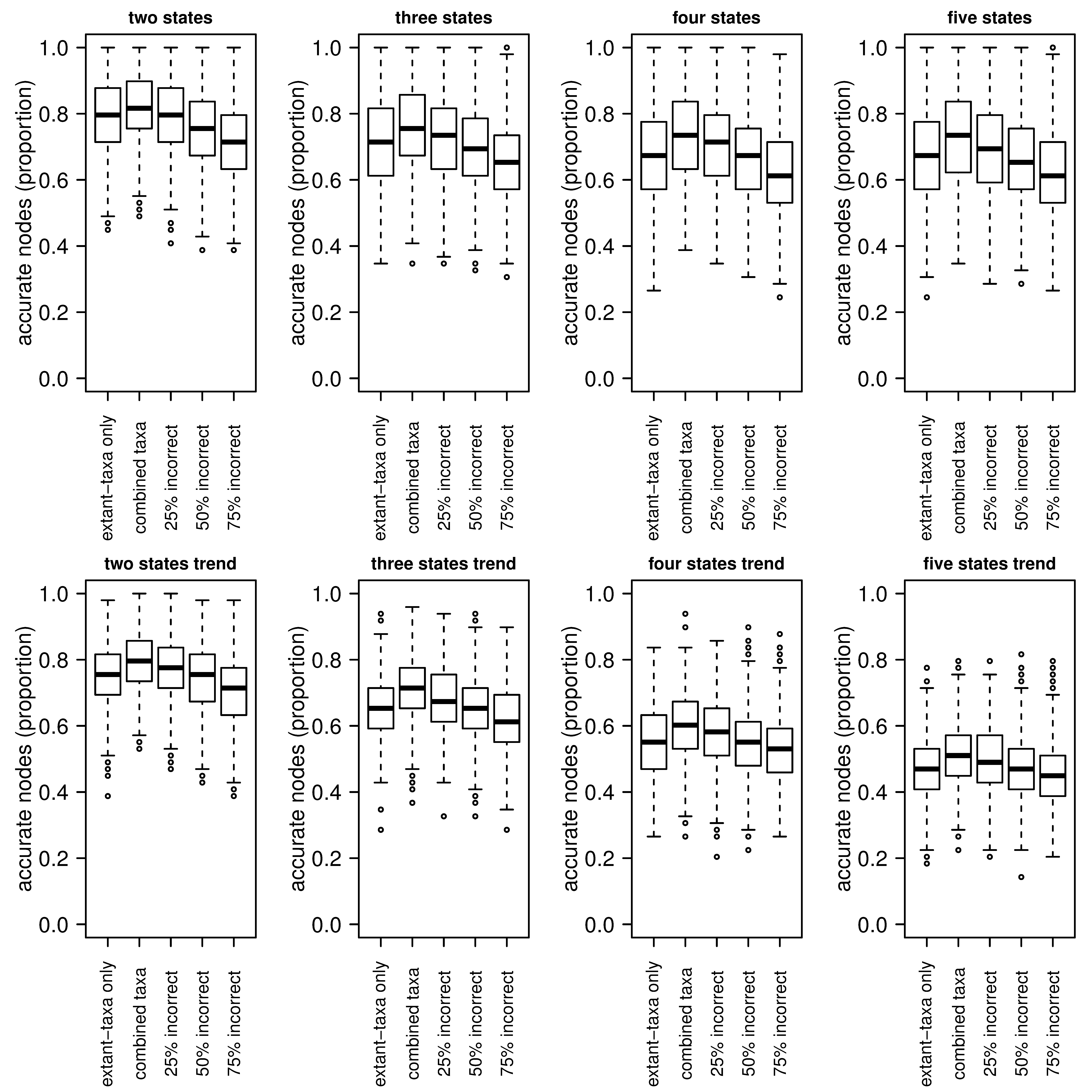


**Supplementary Figure S1.** Node reconstruction on phylogenies with fifty extant species. The accuracy of node reconstructions as shown by the proportion of correctly-reconstructed nodes (the true state has the highest reconstructed marginal likelihood) for all states on the phylogeny with fifty extant species (with both the variable rates and trend datasets).


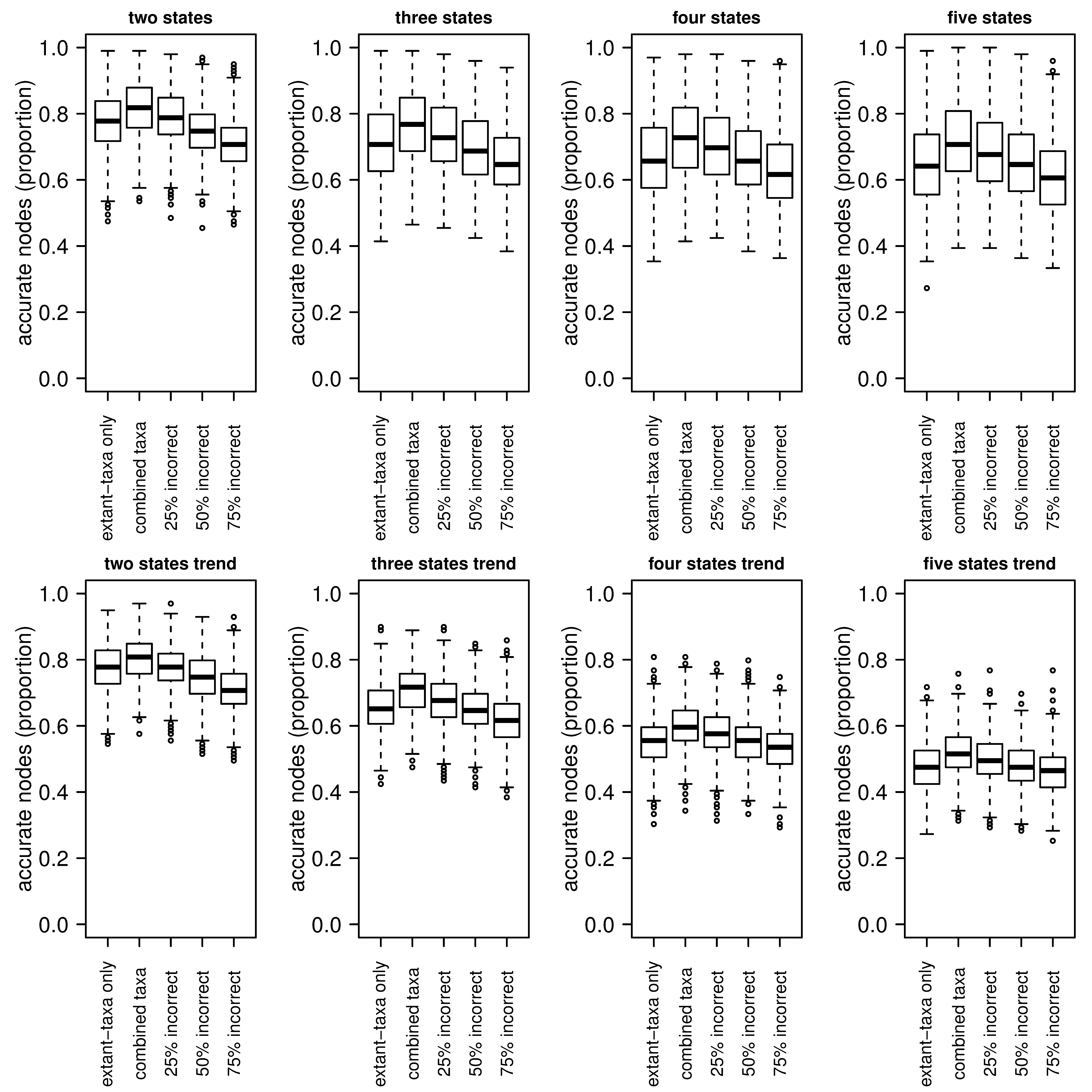


**Supplementary Figure S2.** Node reconstruction on phylogenies with one hundred extant species. The accuracy of node reconstructions as shown by the proportion of correctly-reconstructed nodes (the true state has the highest reconstructed marginal likelihood) for all states on the phylogeny with one hundred extant species (with both the variable rates and trend datasets).


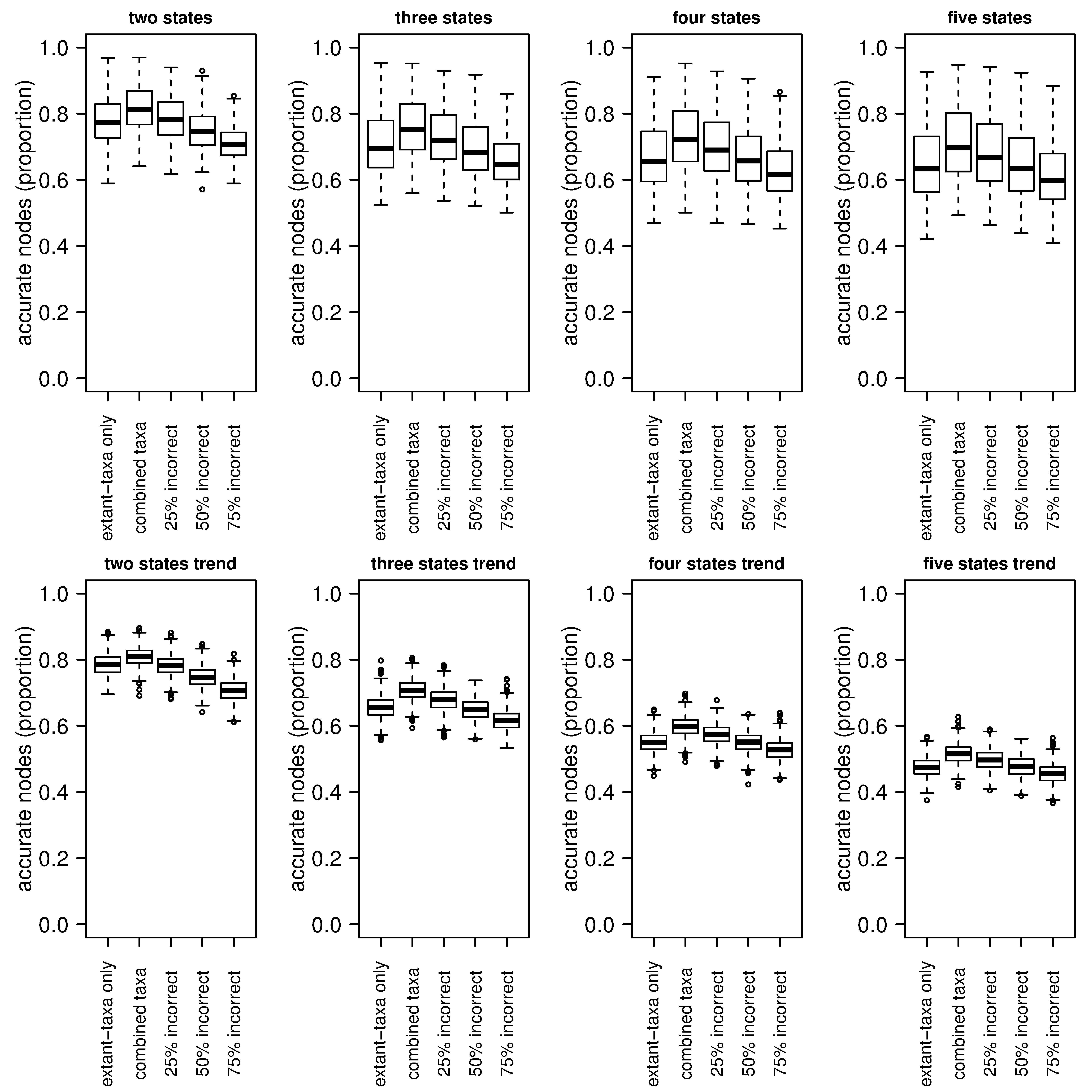


**Supplementary Figure S3.** Node reconstruction on phylogenies with five hundred extant species. The accuracy of node reconstructions as shown by the proportion of correctly-reconstructed nodes (the true state has the highest reconstructed marginal likelihood) for all states on the phylogeny with five hundred extant species (with both the variable rates and trend datasets).


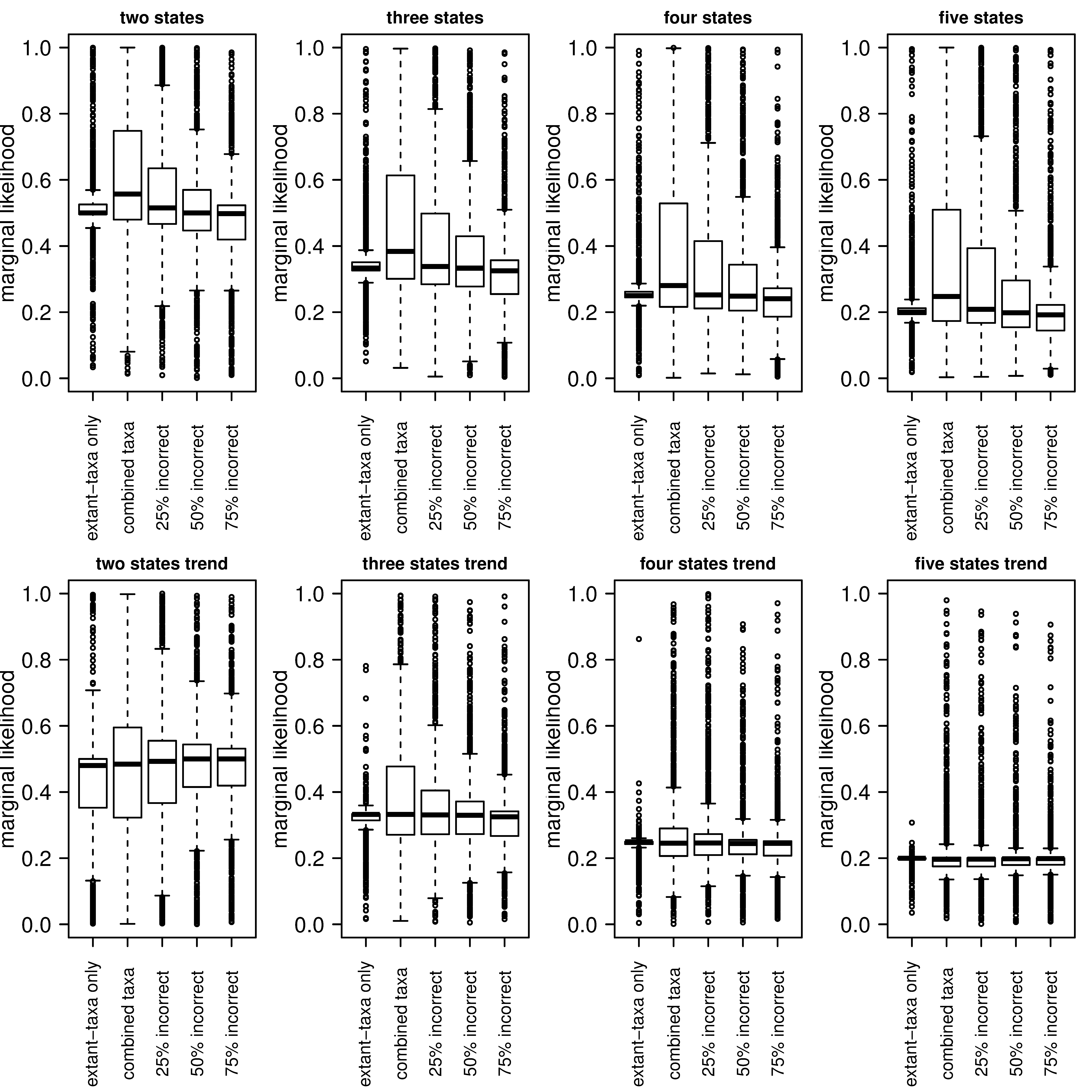


**Supplementary Figure S4.** Marginal likelihood values on the root node on phylogenies with fifty extant species.


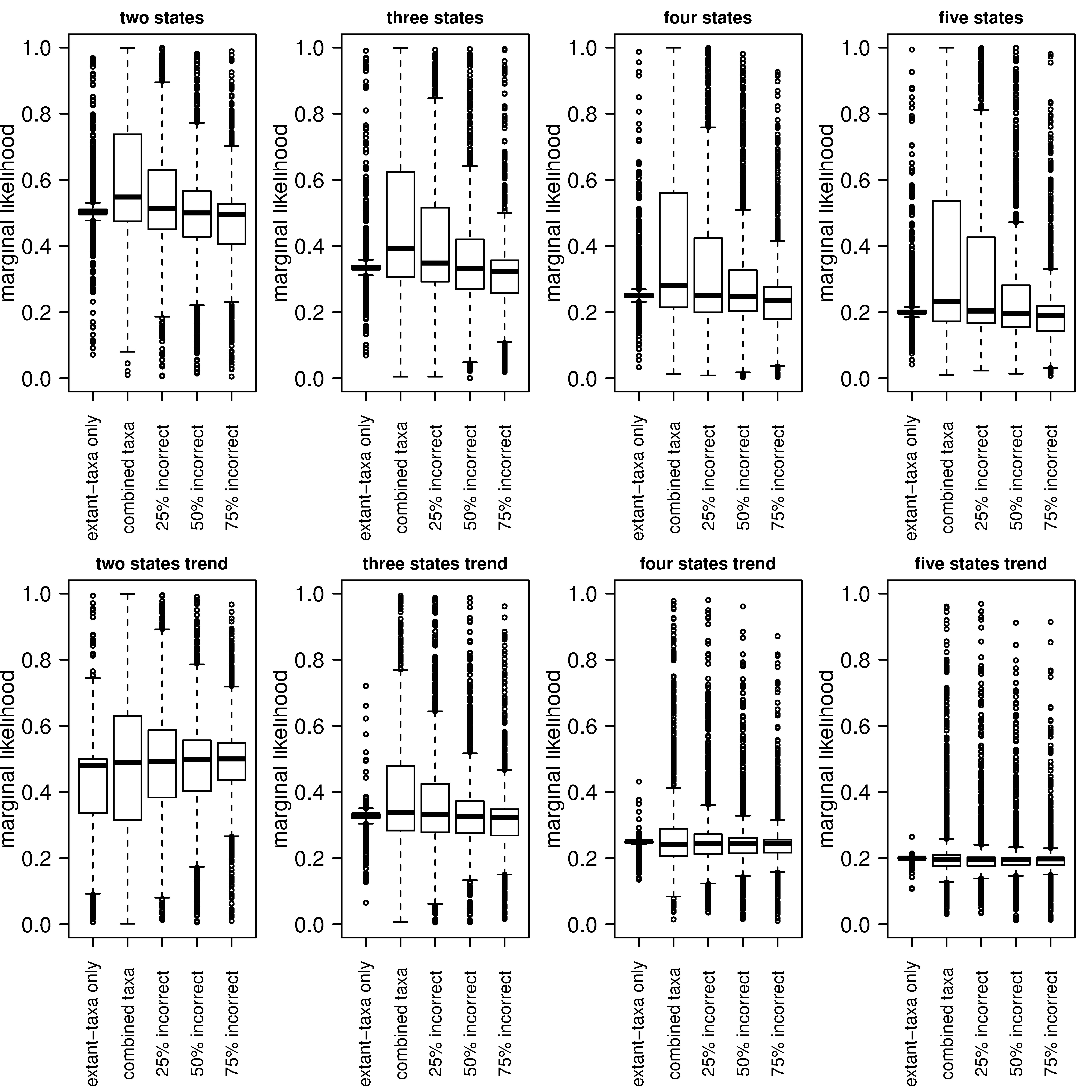


**Supplementary Figure S5.** Marginal likelihood values on the root node on phylogenies with one hundred extant species.


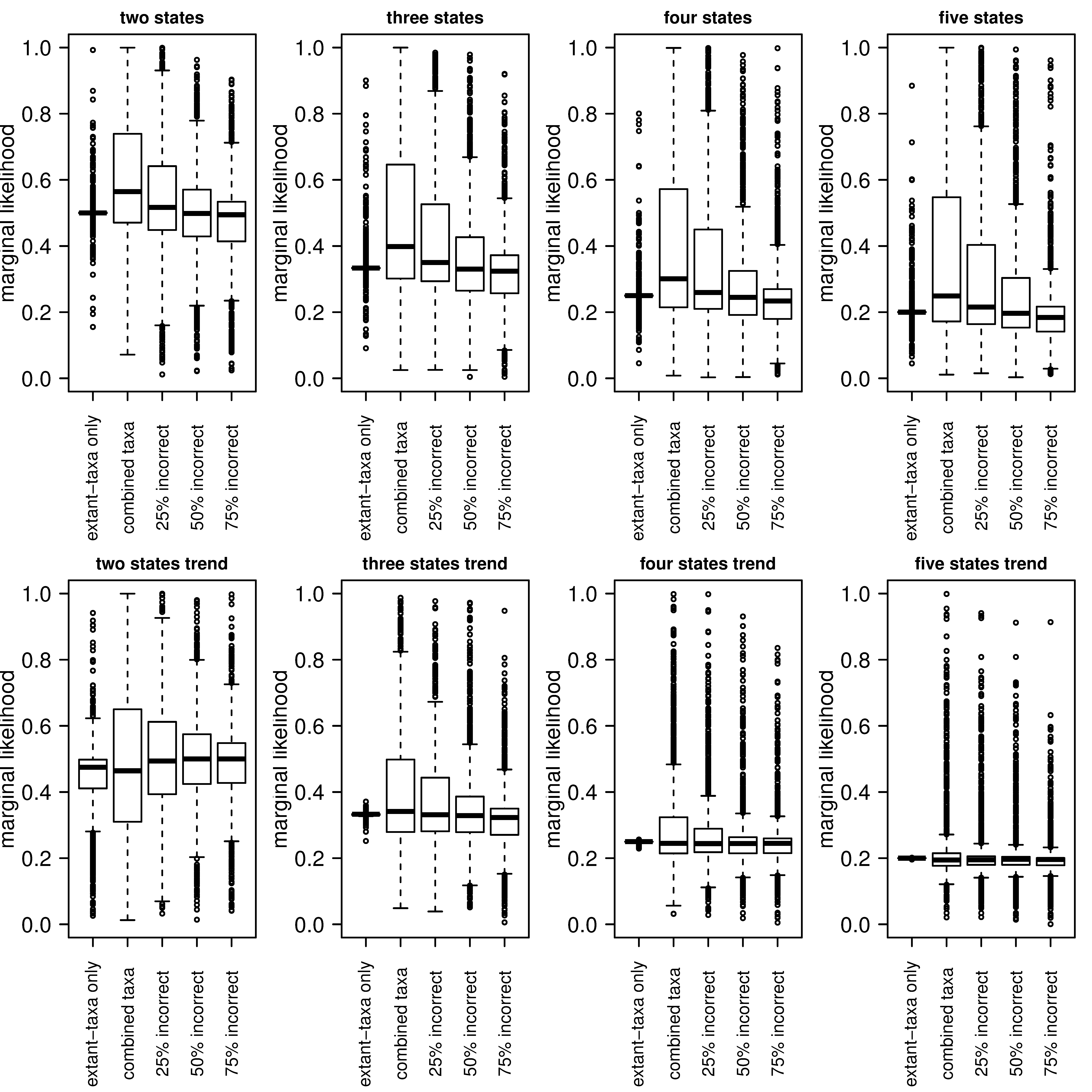


**Supplementary Figure S6.** Marginal likelihood values on the root node on phylogenies with five hundred extant species.
